# Supplementary material for: A PKB-SPEG signaling nexus links insulin resistance with diabetic cardiomyopathy by regulating calcium homeostasis
Source: Nat Commun. 2020 May 4;11:2186. doi: 10.1038/s41467-020-16116-9 (PMC7198626; doi:10.1038/s41467-020-16116-9)
Supplement: Supplementary file 1 — Supplementary Information [file 41467_2020_16116_MOESM1_ESM.pdf]

Supplementary Information for

A PKB-SPEG signaling nexus links insulin resistance with diabetic cardiomyopathy by regulating calcium homeostasis

Quan et al.

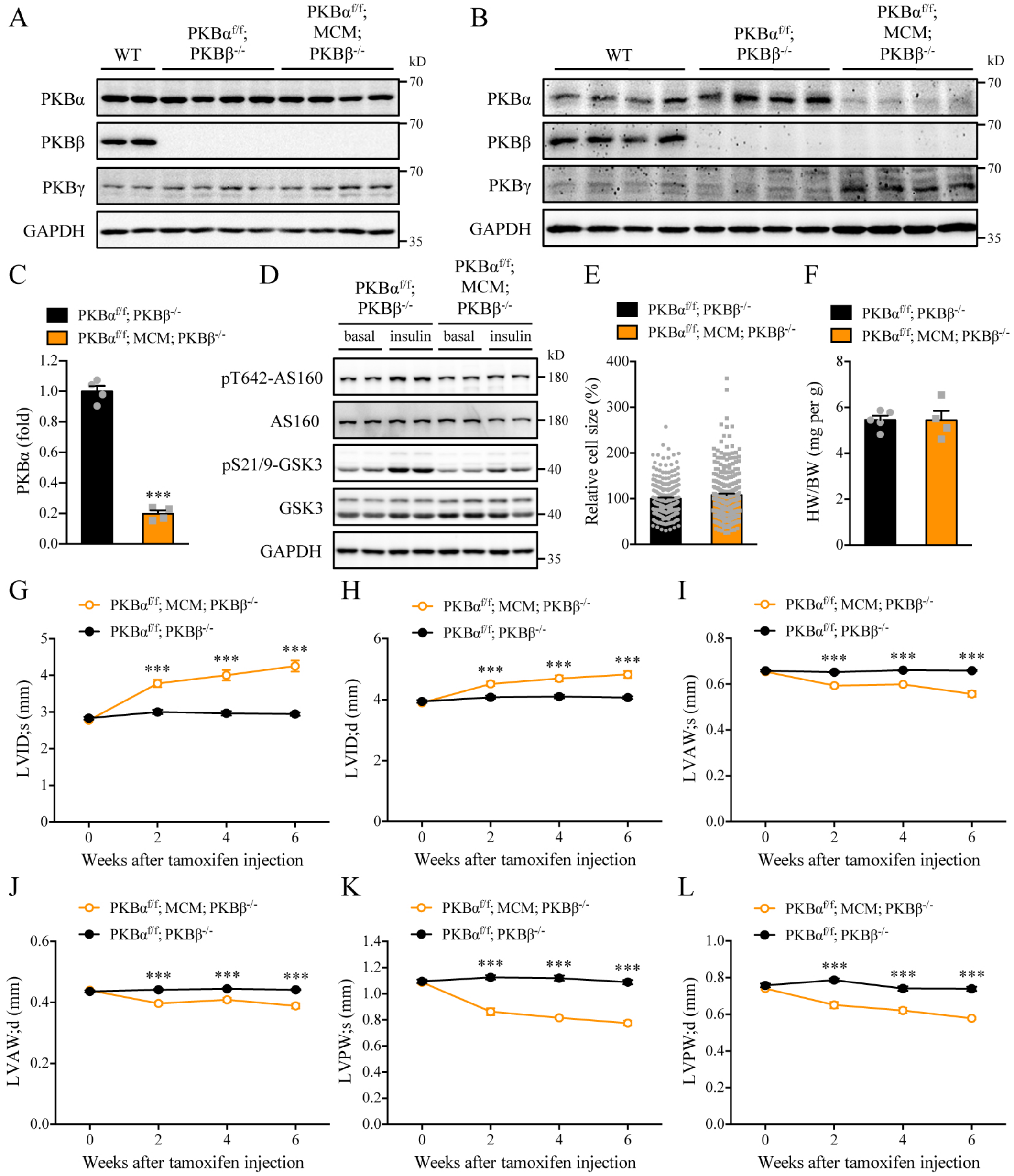

Supplementary Figure 1 Effects of PKBα/β deletion in the heart on cardiac function

A. PKB isoforms in the heart of male PKBα$^{f/f}$;MCM;PKBβ$^{-/-}$ mice before tamoxifen induction. B-C. PKB isoforms in primary cardiomyocytes from the heart of male PKBα$^{f/f}$;MCM;PKBβ$^{-/-}$ mice at 4 weeks after tamoxifen induction. Quantitation of PKBα was shown in C. n = 4. p = 1.54e-6. D. Phosphorylation of AS160 and GSK3 in the heart of male PKBα$^{f/f}$;MCM;PKBβ$^{-/-}$ mice at 6 weeks after tamoxifen induction in response to insulin. E. Relative sizes of cardiomyocytes of male PKBα$^{f/f}$;MCM;PKBβ$^{-/-}$ mice at 4 weeks after tamoxifen induction. n = 240 (PKBα$^{f/f}$;PKBβ$^{-/-}$) and 273 (PKBα$^{f/f}$;MCM;PKBβ$^{-/-}$). p = 0.053. F. Ratio of heart to body-weight in male PKBα$^{f/f}$;MCM;PKBβ$^{-/-}$ mice at 6 weeks after tamoxifen induction. n = 5 (PKBα$^{f/f}$;PKBβ$^{-/-}$) and 4 (PKBα$^{f/f}$;MCM;PKBβ$^{-/-}$). p = 0.978. G-L. Systolic left ventricular diameter (LVID;s) (G), diastolic left ventricular diameter (LVID;d) (H), systolic left ventricular anterior wall (LVAW;s) (I), diastolic left ventricular anterior wall (LVAW;d) (J), systolic left ventricular posterior wall (LVPW;s) (K), and diastolic left ventricular posterior wall (LVPW;d) (L) were measured via echocardiography in male PKBα$^{f/f}$; PKBβ$^{-/-}$ and PKBα$^{f/f}$;MCM;PKBβ$^{-/-}$ mice before and after tamoxifen induction. n = 14 (0-week) and 12 (2-, 4- and 6-week) for PKBα$^{f/f}$;PKBβ$^{-/-}$ mice. n = 12 (0-week), 9 (2-week), 8 (4-week) and 7 (6-week) for PKBα$^{f/f}$;MCM;PKBβ$^{-/-}$ mice. p = 0.245 (0-week), 7.70e-7 (2-week), 4.28e-7 (4-week) and 1.39e-8 (6-week) for LVID;s. p = 0.403 (0-week), 1.10e-4 (2-week), 3.01e-5 (4-week) and 1.09e-6 (6-week) for LVID;d. p = 0.386 (0-week), 9.80e-7 (2-week), 1.21e-9 (4-week) and 1.56e-8 (6-week) for LVAW;s. p = 0.402 (0-week), 7.27e-8 (2-week), 5.20e-6 (4-week) and 7.08e-7 (6-week) for LVAW;d. p = 0.708 (0-week), 5.20e-8 (2-week), 7.97e-10 (4-week) and 7.17e-10 (6-week) for LVPW;s. p = 0.200 (0-week), 4.97e-7 (2-week), 6.99e-6 (4-week) and 1.23e-8 (6-week) for LVPW;d.

Data are given as the mean ± SEM. Statistical analyses were performed using two-sided t-test. Three-asterisk indicates $p < 0.001$. Source data are provided as a Source Data file.

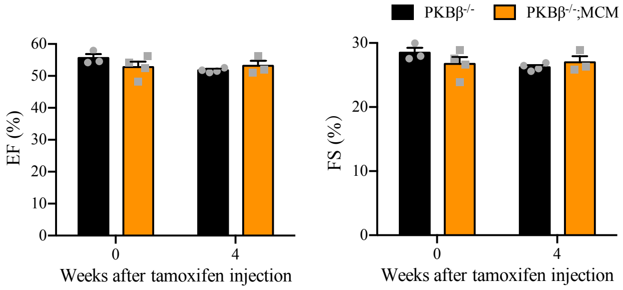

Supplementary Figure 2 Effects of PKBβ deletion in the heart on cardiac function

Ejection fraction (EF) and fractional shortening (FS) in PKBβ$^{-/-}$ and PKBβ$^{-/-}$;MCM mice before and after tamoxifen induction. n = 3 (0-week) and 4 (4-week) for PKBβ$^{-/-}$ mice. n = 4 (0-week) and 3 (4-week) for PKBβ$^{-/-}$;MCM mice.

Data are given as the mean ± SEM. Statistical analyses were performed using two-way ANOVA. No significant difference was detected. Source data are provided as a Source Data file.

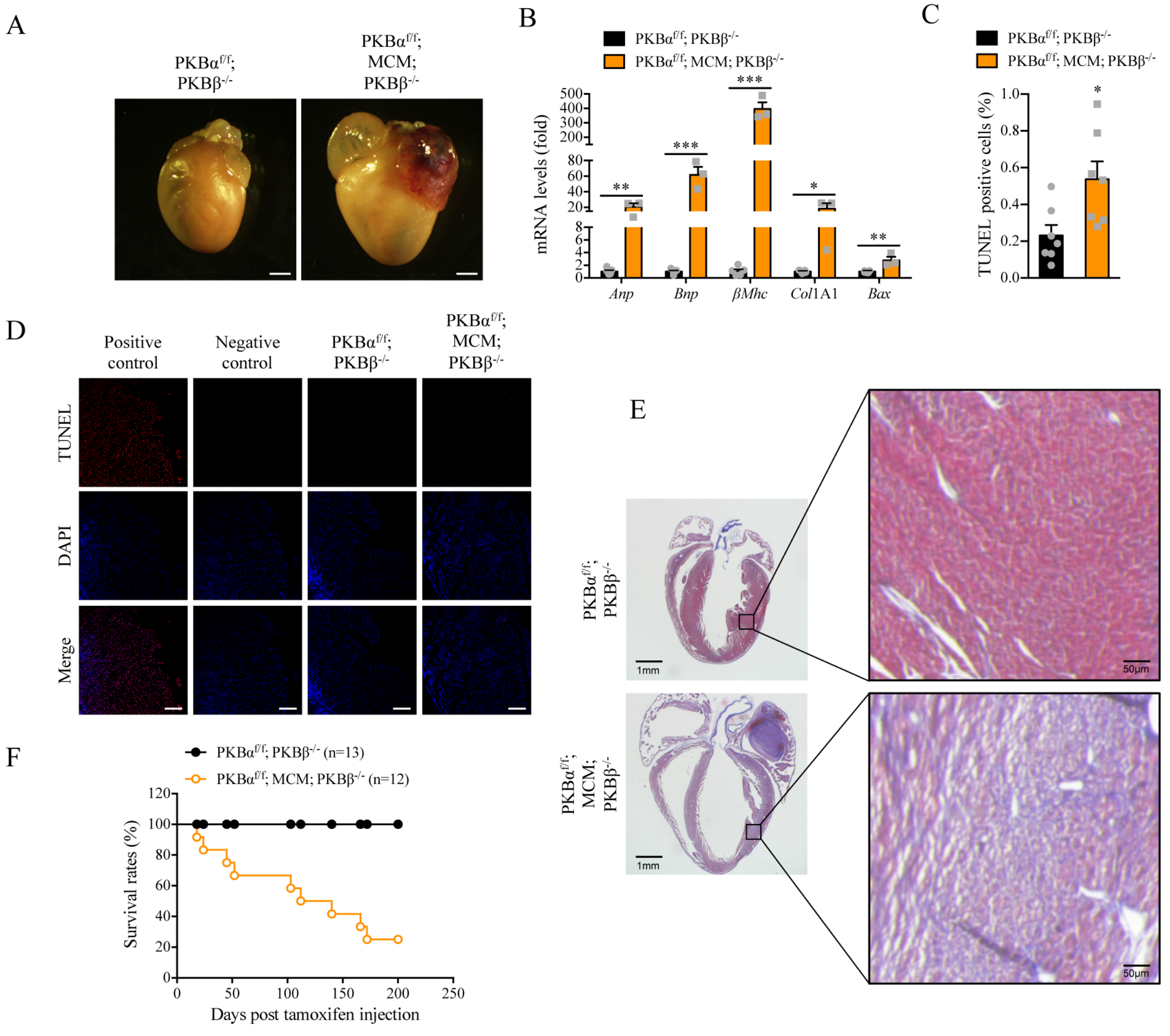

Supplementary Figure 3 Cardiac remodeling in the PKBα$^{f/f}$;MCM;PKBβ$^{-/-}$ mice after tamoxifen induction

A. The hearts from PKBα$^{f/f}$; PKBβ$^{-/-}$ and PKBα$^{f/f}$;MCM;PKBβ$^{-/-}$ mice at 6 weeks after tamoxifen induction. Bars indicate 1 mm in length.

B. Expression of *Anp*, *Bnp*, *βMhc*, *Col*1A1 and *Bax* mRNA in the hearts of male PKBα$^{f/f}$; PKBβ$^{-/-}$ and PKBα$^{f/f}$;MCM;PKBβ$^{-/-}$ mice at 8 weeks after tamoxifen induction. n = 5 (PKBα$^{f/f}$; PKBβ$^{-/-}$) and 3 (PKBα$^{f/f}$;MCM;PKBβ$^{-/-}$). p = 1.01e-3 (*Anp*), 1.95e-4 (*Bnp*), 2.23e-5 (*βMhc*), 1.46e-2 (*Col*1A1), and 4.63e-3 (*Bax*).

C-D. TUNEL staining of the heart sections in the male PKBα$^{f/f}$; PKBβ$^{-/-}$ and PKBα$^{f/f}$;MCM;PKBβ$^{-/-}$ mice at 8 weeks after tamoxifen induction. C, quantitation data. D, representative images. n = 7. Bars indicate 200 µm in length. p = 1.78e-2.

E. Masson's staining of the heart sections in the male PKBα$^{f/f}$; PKBβ$^{-/-}$ and PKBα$^{f/f}$;MCM;PKBβ$^{-/-}$ mice at 6 weeks after tamoxifen induction.

F. Survival rates of the male PKBα$^{f/f}$;PKBβ$^{-/-}$ and PKBα$^{f/f}$;MCM;PKBβ$^{-/-}$ mice after tamoxifen induction. n = 13 (PKBα$^{f/f}$;PKBβ$^{-/-}$) and 12 (PKBα$^{f/f}$;MCM;PKBβ$^{-/-}$).

The data are given as the mean ± SEM. Statistical analyses were carried out using two-sided t-test. One-asterisk indicates $p < 0.05$, two-asterisk indicates $p < 0.01$, and three-asterisk indicates $p < 0.001$. Source data are provided as a Source Data file.

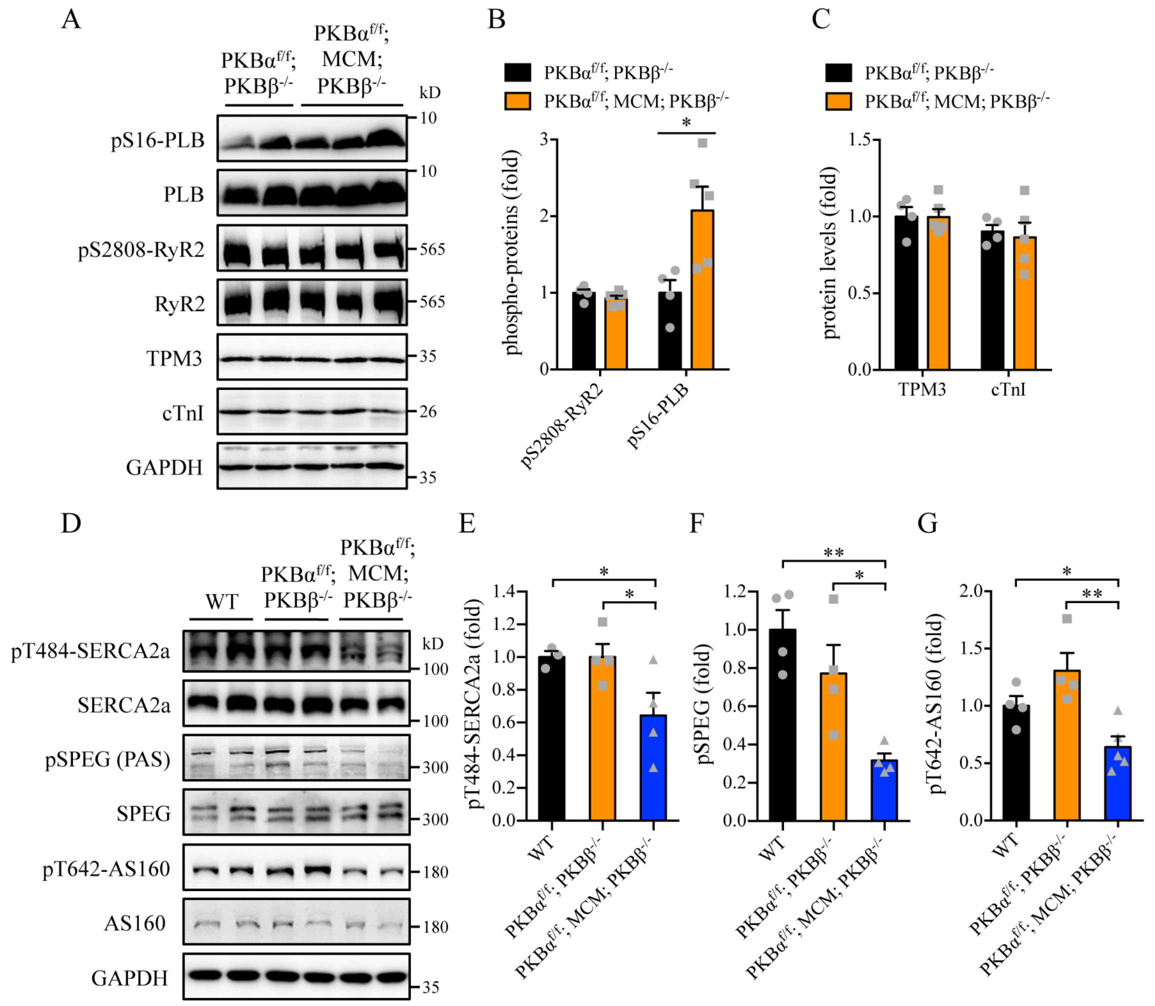

Supplementary Figure 4 Effects of PKBα/β deletion in the heart on phosphorylation of regulators of calcium homeostasis and expression of myofilaments

A-C. Expression and phosphorylation of PLB, RyR2, TPM3 and cTnI in the heart of male PKBα$^{f/f}$;PKBβ$^{-/-}$ and PKBα$^{f/f}$;MCM;PKBβ$^{-/-}$ mice at 4 weeks after tamoxifen induction. A, representative blots. B, quantitation of phosphorylation of PLB and RyR2. p = 0.286 (phosphorylation of RyR2) and 2.66e-2 (phosphorylation of PLB). C, quantitation of expression of TPM3 and cTnI. p = 0.982 (TPM3) and 0.747 (cTnI). n= 4 (PKBα$^{f/f}$;PKBβ$^{-/-}$) and 5 (PKBα$^{f/f}$;MCM;PKBβ$^{-/-}$).

D-G. Phosphorylation of SERCA2a, SPEG, and AS160 in the heart of male PKBα$^{f/f}$; PKBβ$^{-/-}$ and PKBα$^{f/f}$;MCM;PKBβ$^{-/-}$ mice at 4 weeks after tamoxifen induction. D, representative blots. E, quantitation of SERCA2a phosphorylation. n = 3 (WT) and 4 (PKBα$^{f/f}$;PKBβ$^{-/-}$ and PKBα$^{f/f}$;MCM;PKBβ$^{-/-}$). F, quantitation of SPEG phosphorylation. n = 4. G, quantitation of AS160 phosphorylation. n = 4 (WT and PKBα$^{f/f}$;PKBβ$^{-/-}$) and 5 (PKBα$^{f/f}$;MCM;PKBβ$^{-/-}$).

The data are given as the mean ± SEM. Statistical analyses were carried out using two-sided t-test for B-C, and one-way ANOVA for E-G. One-asterisk indicates $p < 0.05$, and two-asterisk indicates $p < 0.01$. Source data are provided as a Source Data file.

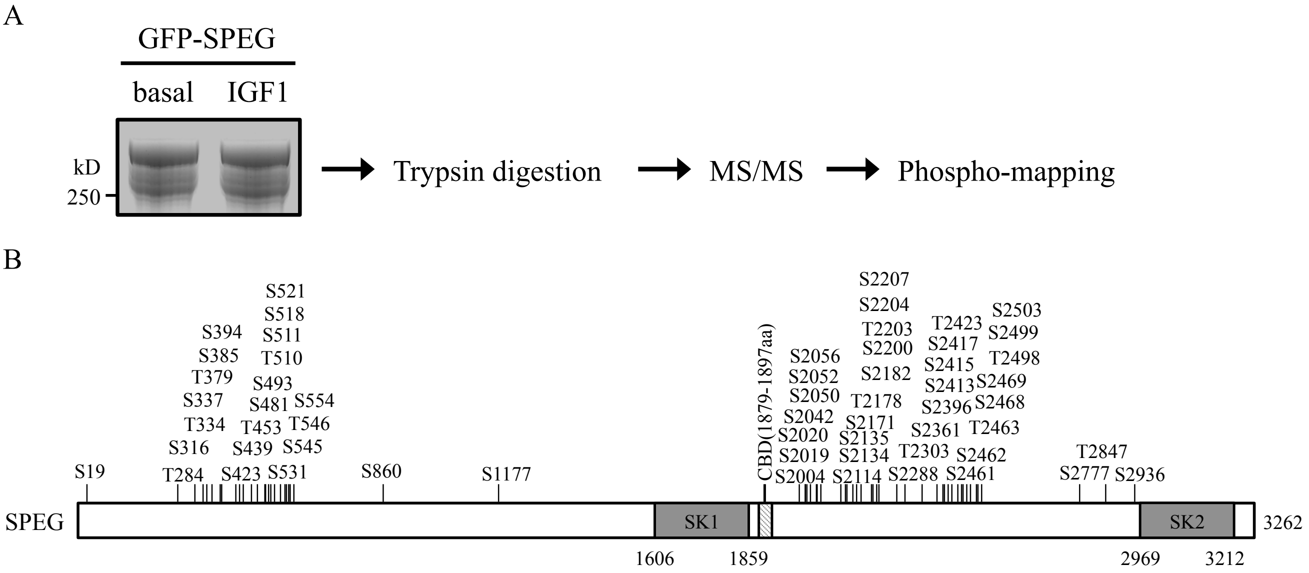

Supplementary Figure 5 Phosphorylation of SPEG in response to stimulation with insulin-like growth factor 1 (IGF1)

GFP-SPEG fusion protein was expressed in HEK293 cells that were stimulated with or without IGF1, and immunoprecipitated from cell lysates using the GFP-binder (A). Phosphorylation sites were identified on immunoprecipitated GFP-SPEG via mass-spectrometry, and listed in Supplementary Table 2 and shown in the diagram (B). Source data are provided as a Source Data file.

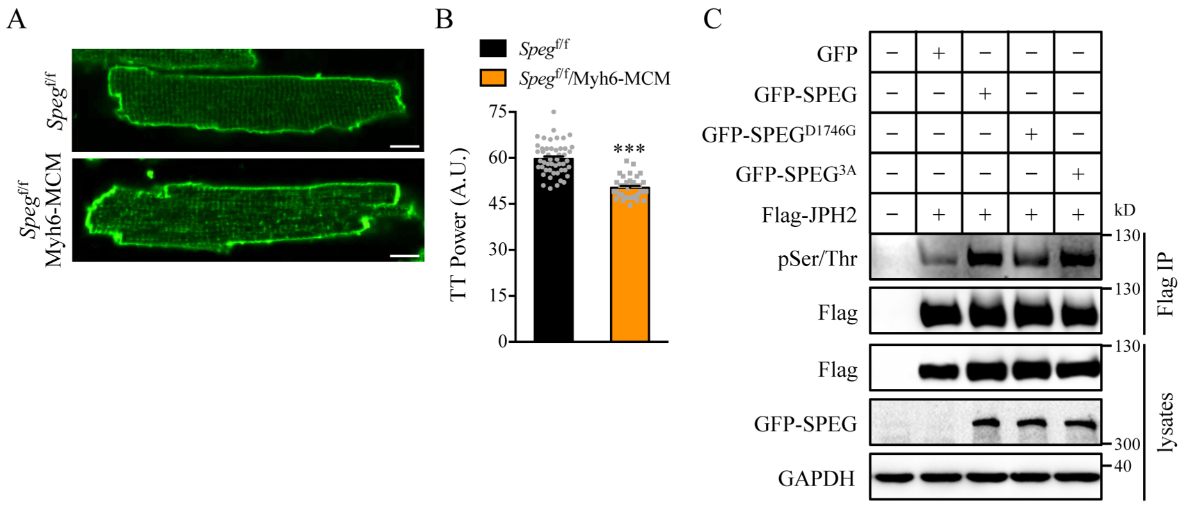

Supplementary Figure 6 T-tubule regularity in *Speg* KO primary cardiomyocytes, and JPH2 phosphorylation by SPEG

A. Representative images for Di-8-ANEPPS staining in primary cardiomyocytes isolated from male *Speg*$^{f/f}$/Myh6-MCM and *Speg*$^{f/f}$ mice 4 weeks post tamoxifen induction. Bars indicate 10 µm in length.

B. Normalized TT power in primary cardiomyocytes isolated from male *Speg*$^{f/f}$/Myh6-MCM and *Speg*$^{f/f}$ mice 4 weeks post tamoxifen induction. A.U., arbitrary unit. n = 50 (*Speg*$^{f/f}$) and 36 (*Speg*$^{f/f}$/Myh6-MCM). p = 5.82e-15.

C. Flag-JPH2 was co-expressed with GFP-SPEG WT or mutant proteins in HEK293 cells. After immunoprecipitated from cell lysates, phosphorylation of Flag-JPH2 was detected using the pSer/Thr antibody.

The data are given as the mean ± SEM. Statistical analysis was carried out using two-sided t-test. Three-asterisk indicates $p < 0.001$. Source data are provided as a Source Data file.

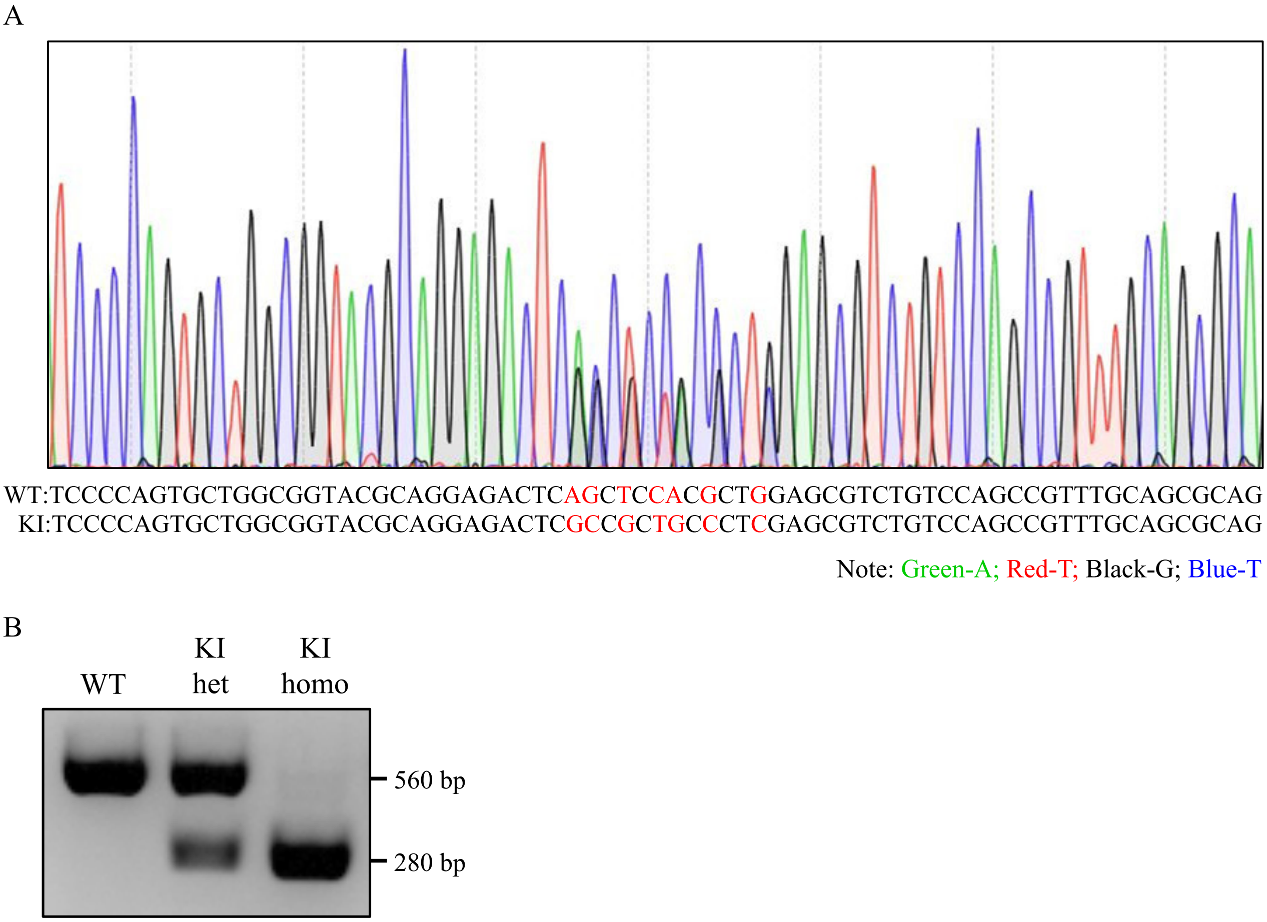

Supplementary Figure 7 Generation and genotyping of the *Speg*[3A] knockin mice

A. Genomic DNA sequences of the WT and knockin *Speg* allele in the heterozygous *Speg*[3A] mice.

B. The *Speg*[3A] mice were genotyped by amplifying the mutated region (562 bp) followed by restriction digestion with *Xho*I (280/282 bp cleaved products for *Speg*[3A] knockins).

Source data are provided as a Source Data file.

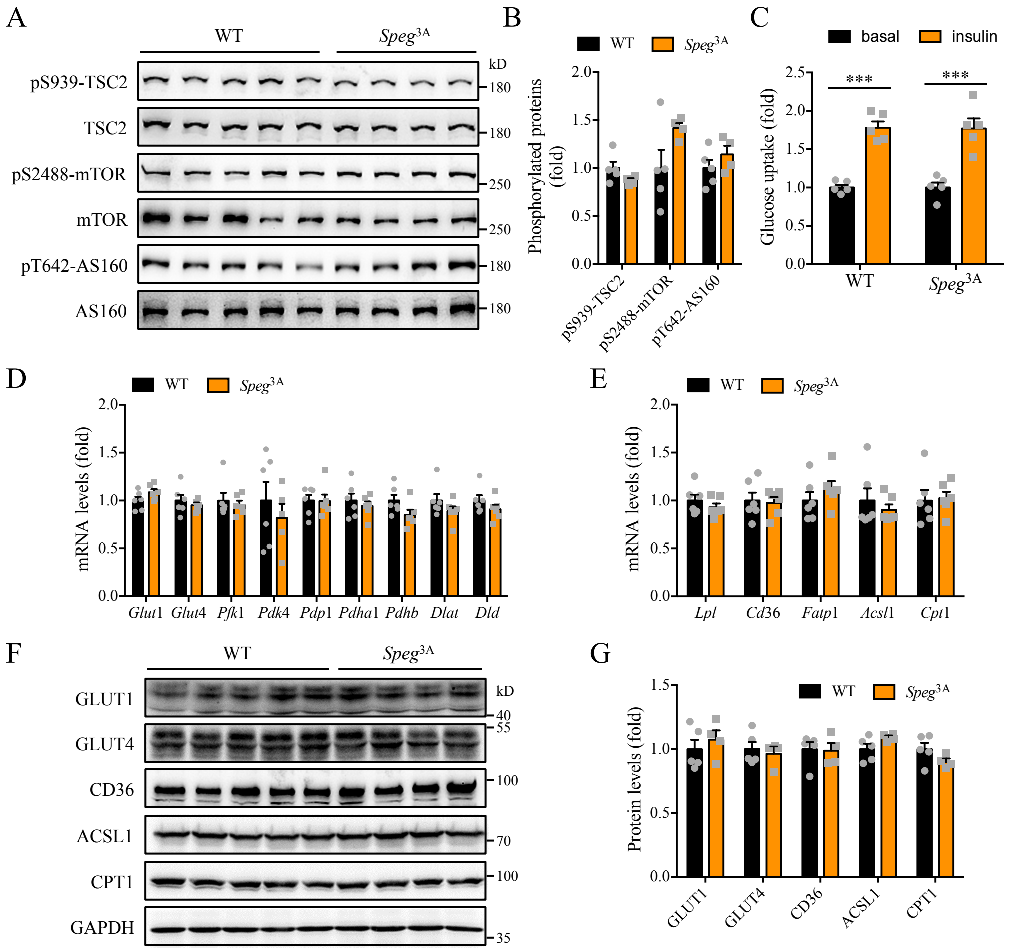

Supplementary Figure 8 Glucose and lipid metabolism in the heart of *Speg*^{3A} knockin mice

A-B. Phosphorylation of TSC2, mTOR and AS160 in the heart of male *Speg*^{3A} mice (7-month-old). Blots shown in A were quantified in B. n = 5 (WT) and 4 (*Speg*^{3A}). p = 0.136 (pS939-TSC2), 0.101 (pS2488-mTOR), and 0.297 (pT642-AS160).

C. Glucose uptake in primary cardiomyocytes isolated from the *Speg*^{3A} mice. n = 5.

D. mRNA levels of key genes for glucose metabolism in the heart of male *Speg*^{3A} mice (2-month-old). n = 6 for all the samples except for *Pdk*4 of *Speg*^{3A} where n = 5. p = 0.113 (*Glut*1), 0.458 (*Glut*4), 0.641 (*Pfk*1), 0.489 (*Pdk*4), 0.957 (*Pdp*1), 0.527 (*Pdha*1), 0.089 (*Pdhb*), 0.215 (*Dlat*) and 0.239 (*Dld*).

E. mRNA levels of key genes for lipid metabolism in the heart of male *Speg*^{3A} mice (2-month-old). n = 6. p = 0.350 (*Lpl*), 0.811 (*Cd*36), 0.330 (*Fatp*1), 0.494 (*Acsl*1), and 0.853 (*Cpt*1).

F-G. Protein expression of key enzymes for glucose and lipid metabolism in the heart of male *Speg*^{3A} mice (7-month-old). Blots shown in F were quantified in G. n = 5 (WT) and 4 (*Speg*^{3A}). p = 0.496 (GLUT1), 0.657 (GLUT4), 0.886 (CD36), 0.112 (ACSL1) and 0.147 (CPT1).

The data are given as the mean ± SEM. Statistical analyses were carried out using two-sided t-test for B, D, E, and G, and two-way ANOVA for C. Three-asterisk indicates $p < 0.001$. Source data are provided as a Source Data file.

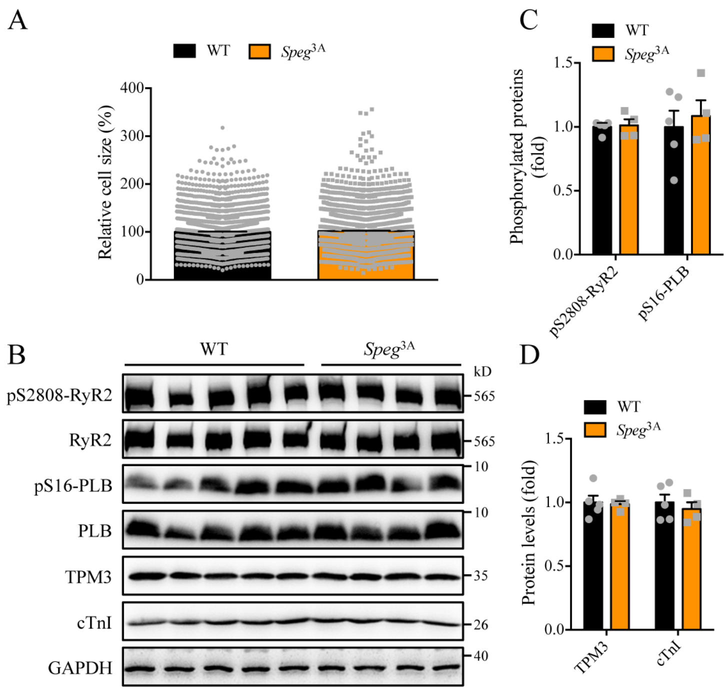

Supplementary Figure 9 Cardiomyocyte sizes, regulators of calcium homeostasis and myofilaments in the heart of *Speg*^{3A} knockin mice

A. The relative sizes of cardiomyocytes of male *Speg*^{3A} mice (3-month-old). n = 2507 (WT) and 2954 (*Speg*^{3A}). $p = 0.118$.

B-D. Expression and phosphorylation of PLB, RyR2, TPM3 and cTnI in the heart of male *Speg*^{3A} mice (7-month-old). B, representative blots. C, quantitation of phosphorylation of PLB and RyR2. $p = 0.807$ (pS2808-RyR2) and 0.646 (pS16-PLB). C, quantitation of expression of TPM3 and cTnI. $p = 0.842$ (TPM3) and 0.566 (cTnI). n= 5 (WT) and 4 (*Speg*^{3A}).

The data are given as the mean ± SEM. Statistical analyses were carried out using two-sided *t*-test. Source data are provided as a Source Data file.

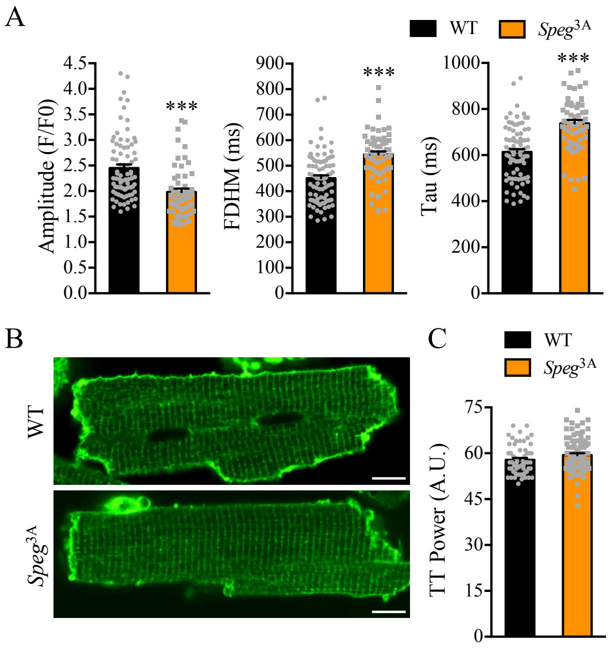

Supplementary Figure 10 Calcium transients and T-tubule regularity in *Speg*^{3A} primary cardiomyocytes

A. Calcium transients elicited by electrical stimulation in primary cardiomyocytes isolated from the WT and *Speg*^{3A} knockin mice (7-month-old). Amplitudes, full duration at half maximum (FDHM) and time constant Tau of calcium transients were quantified from 80 WT cells and 57 *Speg*^{3A} knockin cells. p = 5.29e-6 (amplitude), 1.56e-7 (FDHM), and 9.52e-9 (Tau).

B. Representative images for Di-8-ANEPPS staining in primary cardiomyocytes isolated from male *Speg*^{3A} knockin mice and WT littermates (8-week-old). Bars indicate 10 µm in length.

C. Normalized TT power in primary cardiomyocytes isolated from male *Speg*^{3A} knockin mice and WT littermates (8-week-old). A.U., arbitrary unit. n = 60 (WT) and 88 (*Speg*^{3A}). p = 0.076.

The data are given as the mean ± SEM. Statistical analyses were carried out using two-sided t-test. Three-asterisk indicates $p < 0.001$. Source data are provided as a Source Data file.

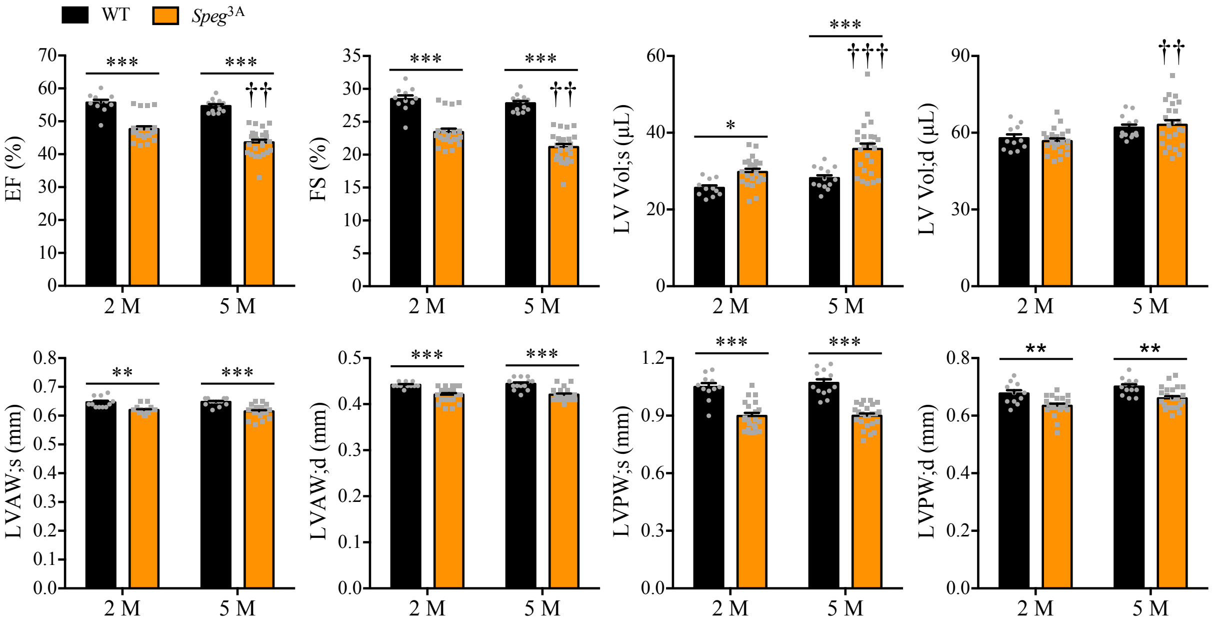

Supplementary Figure 11 Echocardiographic parameters of the female wild-type and *Speg*^{3A} knockin hearts

Echocardiography was performed on the anaesthetized female *Speg*^{3A} knockin mice and wild-type littermates at age of 2 and 5 months to measure EF, FS, LV Vol;s, LV Vol;d, LVAW;s, LVAW;d, LVPW;s, and LVPW;d. n = 11 (2M) and 13 (5M) for WT mice. n = 21 (2M) and 24 (5M) for *Speg*^{3A} knockin mice.

The data are given as the mean ± SEM. Statistical analyses were carried out using two-way ANOVA. One-asterisk (WT vs *Speg*^{3A}) indicates $p < 0.05$. Two-asterisk (WT vs *Speg*^{3A}), and two-dagger (*Speg*^{3A} 2M vs *Speg*^{3A} 5M) indicate $p < 0.01$. Three-asterisk (WT vs *Speg*^{3A}), and three-dagger (*Speg*^{3A} 2M vs *Speg*^{3A} 5M) indicate $p < 0.001$. Source data are provided as a Source Data file.

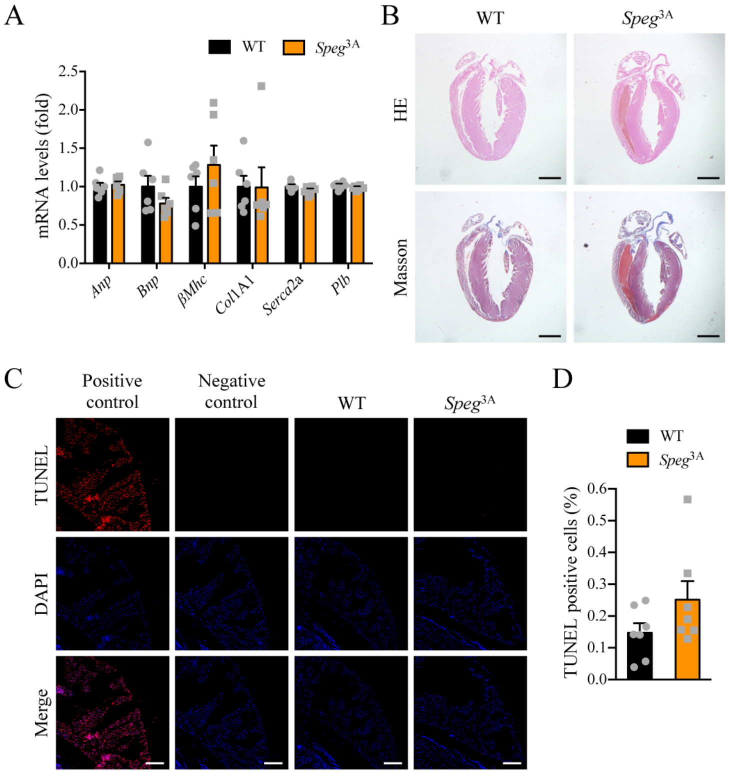

Supplementary Figure 12 Cardiac remodeling in the heart of *Speg*^{3A} knockin mice

A. Expression of *Anp*, *Bnp*, *βMhc*, *Col*1A1, *Serca*2a and *Plb* mRNA in the hearts of male *Speg*^{3A} mice (3-month-old). n = 6. *p* = 0.715 (*Anp*), 0.195 (*Bnp*), 0.349 (*βMhc*), 0.968 (*Col*1A1), 0.094 (*Serca*2a) and 0.256 (*Plb*).

B. Masson's staining of the heart sections in the male *Speg*^{3A} mice (9-month-old). Bars indicate 1 mm in length.

C-D. TUNEL staining of the heart sections in the male *Speg*^{3A} mice (9-month-old). G, representative images. H, quantitation data. n = 7. *p* = 0.140. Bars indicate 200 μm in length.

The data are given as the mean ± SEM. Statistical analyses were carried out using two-sided *t*-test. Source data are provided as a Source Data file.
