# Supplementary material for: A PKB-SPEG signaling nexus links insulin resistance with diabetic cardiomyopathy by regulating calcium homeostasis
Source: Nat Commun. 2020 May 4;11:2186. doi: 10.1038/s41467-020-16116-9 (PMC7198626; doi:10.1038/s41467-020-16116-9)
Supplement: Supplementary file 3 — Description of Additional Supplementary Files [file 41467_2020_16116_MOESM3_ESM.docx]

**File Name: Supplementary Data 1**

**Description:** Proteins identified in the PAS immunoprecipitates from mouse heart lysates. Phosphorylated proteins were immunoprecipitated using the PAS antibody from heart lysates of the mice that were intraperitoneally injected with or without insulin for 20 min after an overnight fast. The immunoprecipitated proteins were electrophoretically separated in NuPAGE® Bis-Tris gels and stained with Coomassie dye (see Fig. 2B). The protein bands were excised, digested with trypsin and identified via mass-spectrometry. Proteins that had at least two unique peptides identified and total Mascot ion scores over 50 were considered as positive hits. A number of proteins had ion scores that were higher (>1.3 fold) in the PAS-captured samples from the hearts of insulin-treated mice than from fasted animals, which are highlighted in red including known PKB susbstrates such as RalGAPα1, RalGAPα2, AS160 (also known as TBC1D4), TSC2 (also known as Tuberin), WNK1 and NDRG1.

**File Name: Supplementary Data 2**

**Description:** Phosphopeptides identified on SPEG. GFP-SPEG proteins were immunoprecipitated using the GFP-binder from cell lysates that were stimulated with or without IGF1 (Supple. Fig. 5A). Phosphopeptides were detected on the immunoprecipitated GFP-SPEG proteins via mass spectrometry. The phosphorylation sites were underlined.

**File Name: Supplementary Data 3**

**Description:** The list of commercial antibodies and resins used in this study
